# Sex and APOE genotype specific brain regional vulnerability to Alzheimer's Disease

**DOI:** 10.1007/s11357-025-02089-4

**Published:** 2026-02-18

**Authors:** Qi Zeng, Minghui Wang, Erming Wang, Xianxiao Zhou, Peng Xu, Vahram Haroutunian, Dongming Cai, Bin Zhang, Members of the Alzheimers Disease Neuroimaging Initiative, Michael Weiner, Paul Aisen, Ronald Petersen, Clifford R. Jack, William Jagust, Susan Landau, Monica Rivera-Mindt, Ozioma Okonkwo, Leslie M. Shaw, Edward B. Lee, W. Toga, Laurel Beckett, Danielle Harvey, Robert C. Green, Andrew J. Saykin, Kwangsik Nho, Richard J. Perrin, Duygu Tosun, Pallavi Sachdev, Erin Drake, Tom Montine, Rachel Nosheny, Diana Truran Sacrey, Juliet Fockler, Melanie J. Miller, Catherine Cat Conti, Winnie Kwang, Chengshi Jin, Adam Diaz, Miriam Ashford, Derek Flenniken, Adrienne Kormos, Paul Aisen, Michael Rafii, Rema Raman, Gustavo Jimenez, Michael Donohue, Jennifer Salazar, Andrea Fidell, Virginia Boatwright, Justin Robison, Caileigh Zimmerman, Yuliana Cabrera, Sarah Walter, Taylor Clanton, Elizabeth Shaffer, Caitlin Webb, Lindsey Hergesheimer, Stephanie Smith, Sheila Ogwang, Olusegun Adegoke, Payam Mahboubi, Erin Drake, Jeremy Pizzola, Cecily Jenkins, Laurel Beckett, Danielle Harvey, Michael Donohue, Naomi Saito, Adam Diaz, Kedir Adem Hussen, Ozioma Okonkwo, Monica Rivera-Mindt, Hannatu Amaza, Mai Seng Thao, Shaniya Parkins, Omobolanle Ayo, Matt Glittenberg, Isabella Hoang, Kaori Kubo Germano, Joe Strong, Trinity Weisensel, Fabiola Magana, Lisa Thomas, Vanessa Guzman, Adeyinka Ajayi, Joseph Di Benedetto, Sandra Talavera, Joel Felmlee, Nick C. Fox, Paul Thompson, Charles DeCarli, Arvin Forghanian-Arani, Bret Borowski, Calvin Reyes, Caitie Hedberg, Chad Ward, Christopher Schwarz, Denise Reyes, Jeff Gunter, John Moore-Weiss, Kejal Kantarci, Leonard Matoush, Matthew Senjem, Prashanthi Vemuri, Robert Reid, Ian Malone, Sophia I. Thomopoulos, Talia M. Nir, Neda Jahanshad, Alexander Knaack, Evan Fletcher, Danielle Harvey, Duygu Tosun-Turgut, Stephanie Rossi Chen, Mark Choe, Karen Crawford, Paul A. Yushkevich, Sandhitsu Das, Sandra Talavera, Joel Felmlee, Nick C. Fox, Paul Thompson, Charles DeCarli, Arvin Forghanian-Arani, Bret Borowski, Calvin Reyes, Caitie Hedberg, Chad Ward, Christopher Schwarz, Denise Reyes, Jeff Gunter, John Moore-Weiss, Kejal Kantarci, Leonard Matoush, Matthew Senjem, Prashanthi Vemuri, Robert Reid, Ian Malone, Sophia I. Thomopoulos, Talia M. Nir, Neda Jahanshad, Alexander Knaack, Evan Fletcher, Danielle Harvey, Duygu Tosun-Turgut, Stephanie Rossi Chen, Mark Choe, Karen Crawford, Paul A. Yushkevich, Sandhitsu Das, William Jagust, Susan Landau, Robert A. Koeppe, Gil Rabinovici, Victor Villemagne, Brian LoPresti, Richard J. Perrin, John Morris, Erin Franklin, Haley Bernhardt, Nigel J. Cairns, Lisa Taylor-Reinwald, Leslie Shaw, Edward B. Lee, Virginia M. Y. Lee, Magdalena Korecka, Magdalena Brylska, Yang Wan, J. Q. Trojanowki, Arthur W. Toga, Karen Crawford, Scott Neu, Andrew J. Saykin, Kwangsik Nho, Tatiana M. Foroud, Taeho Jo, Shannon L. Risacher, Hannah Craft, Liana G. Apostolova, Kelly Nudelman, Kelley Faber, Zoë Potter, Kaci Lacy, Rima Kaddurah-Daouk, Li Shen, David Soleimani-Meigooni, Renaud La Joie, Konstantinos Chiotis, Maison Abu Raya, Agathe Vrillon, Charles Windon, Julien Lagarde, Zoe Lin, Aidyn Rose Hills, Jason Karlawish, Claire Grill, Emily Largent, Kristin Harkins, Michael W. Weiner, Leon Thal, Zaven Kachaturian, Richard Frank, Peter J. Snyder, Neil Buckholtz, John K. Hsiao, Laurie Ryan, Susan Molchan, Zaven Khachaturian, Maria Carrillo, William Potter, Lisa Barnes, Marie Bernard, Hector González, Carole Ho, Jonathan Jackson, Eliezer Masliah, Donna Masterman, Ozioma Okonkwo, Richard Perrin, Laurie Ryan, Nina Silverberg, Lisa Silbert, Jeffrey Kaye, Sylvia White Salazar, Aimee Pierce, Amy Thomas, Tera Clay, Daniel Schwartz, Gillian Devereux, Janet Janae Taylor, Jennifer Ryan, Mike Nguyen, Nguyen DeCapo, Yanan Shang, Lon Schneider, Cynthia Munoz, Diana Ferman, Carlota Conant, Katherin Martin, Kristin Oleary, Sonia Pawluczyk, Elizabeth Trejo, Karen Dagerman, Liberty Teodoro, Mauricio Becerra, Madiha Fairooz, Sonia Garrison, Julia Boudreau, Yair Avila, James Brewer, Aaron Jacobson, Antonio Gama, Chi Kim, Emily Little, Jennifer Frascino, Nichol Ferng, Socorro Trujillo, Judith Heidebrink, Robert Koeppe, Steven MacDonald, Dariya Malyarenko, Jaimie Ziolkowski, James O’Connor, Nicole Robert, Suzan Lowe, Virginia Rogers, Ronald Petersen, Barbara Hackenmiller, Bradley Boeve, Colleen Albers, Connie Kreuger, David Jones, David Knopman, Hugo Botha, Jessica Magnuson, Jonathan Graff-Radford, Kerry Crawley, Michael Schumacher, Sanna McKinzie, Steven Smith, Tascha Helland, Val Lowe, Vijay Ramanan, Valory Pavlik, Jacob Faircloth, Jeffrey Bishop, Jessica Nath, Maria Chaudhary, Maria Kataki, Melissa Yu, Nathiel Pacini, Randall Barker, Regan Brooks, Ruchi Aggarwal, Lawrence Honig, Yaakov Stern, Akiva Mintz, Jonathan Cordona, Michelle Hernandez, Justin Long, Abbey Arnold, Alex Groves, Anna Middleton, Blake Vogler, Cierra McCurry, Connie Mayo, Cyrus Raji, Fatima S. Amtashar, Heather Klemp, Heather Nicole Elmore, James Ruszkiewicz, Jasmina Kusuran, Jasmine Stewart, Jennifer Horenkamp, Julia Greeson, Kara Wever, Katie Vo, Kelly Larkin, Lesley Rao, Lisa Schoolcraft, Lora Gallagher, Madeline Paczynski, Maureen McMillan, Michael Holt, Nicole Gagliano, Rachel Henson, Renee LaBarge, Robert Swarm, Sarah Munie, Serena Cepeda, Stacey Winterton, Stephen Hegedus, TaNisha Wilson, Tanya Harte, Zach Bonacorsi, David Geldmacher, Amber Watkins, Brandi Barger, Bryan Smelser, Charna Bates, Cynthia Stover, Emily McKinley, Gregory Ikner, Haley Hendrix, Harold Matthew Cooper, Jennifer Mahaffey, Lindsey Booth Robbins, Loren Brown Ashley, Marissa Natelson-Love, Princess Carter, Veronika Solomon, Hillel Grossman, Alexandra Groome, Allison Ardolino, Anthony Kaplan, Faye Sheppard, Genesis Burgos-Rivera, Gina Garcia-Camilo, Joanne Lim, Judith Neugroschl, Kimberly Jackson, Kirsten Evans, Laili Soleimani, Mary Sano, Nasrin Ghesani, Sarah Binder, Xiomara Mendoza Apuango, Ajay Sood, Amelia Troutman, Kimberly Blanchard, Arlene Richards, Grace Nelson, Kirsten Hendrickson, Erin Yurko, Jamie Plenge, Victoria Rufo, Raj Shah, Ranjan Duara, Brendan Lynch, Cesar Chirinos, Christine Dittrich, Debbie Campbell, Diego Mejia, Gilberto Perez, Helena Colvee, Joanna Gonzalez, Josalen Gondrez, Joshua Knaack, Mara Acevedo, Maria Cereijo, Maria Greig-Custo, Michelle Villar, Morris Wishnia, Sheryl Detling, Warren Barker, Marilyn Albert, Abhay Moghekar, Barbara Rodzon, Corey Demsky, Gregory Pontone, Jim Pekar, Leonie Farrington, Martin Pomper, Nicole Johnson, Tolulope Alo, Martin Sadowski, Anaztasia Ulysse, Arjun Masurkar, Brittany Marti, David Mossa, Emilie Geesey, Emily Petrocca, Evan Schulze, Jennifer Wong, Joseph Boonsiri, Sunnie Kenowsky, Tatianne Martinez, Veronica Briglall, P. Murali Doraiswamy, Adaora Nwosu, Alisa Adhikari, Cammie Hellegers, Jeffrey Petrella, Olga James, Terence Wong, Thomas Hawk, Sanjeev Vaishnavi, Hannah McCoubrey, Ilya Nasrallah, Rachel Rovere, Jeffrey Maneval, Elizabeth Robinson, Francisco Rivera, Jade Uffelman, Martha Combs, Patricia O’Donnell, Sara Manning, Richard King, Alayne Nieto, Amanda Glueck, Anjana Mandal, Audrie Swain, Bethanie Gamble, Beverly Meacham, Denece Forenback, Dorothy Ross, Elizabeth Cheatham, Ellen Hartman, Gary Cornell, Jordan Harp, Laura Ashe, Laura Goins, Linda Watts, Morgan Yazell, Prabin Mandal, Regan Buckler, Sylvia Vincent, Triana Rudd, Oscar Lopez, Ann Arlene Malia, Caitlin Chiado, Cary Zik, James Ruszkiewicz, Kathleen Savage, Linda Fenice, MaryAnn Oakley, Paige C. Tacey, Sarah Berman, Sarah Bowser, Stephen Hegedus, Xanthia Saganis, Anton Porsteinsson, Abigail Mathewson, Asa Widman, Bridget Holvey, Emily Clark, Esmeralda Morales, Iris Young, James Ruszkiewicz, Kevin Hopkins, Kimberly Martin, Nancy Kowalski, Rebecca Hunt, Roberta Calzavara, Russell Kurvach, Stephen D’Ambrosio, Gaby Thai, Beatriz Vides, Brigit Lieb, Catherine McAdams-Ortiz, Cyndy Toso, Ivan Mares, Kathryn Moorlach, Luter Lui, Maria Corona, Mary Nguyen, Melanie Tallakson, Michelle McDonnell, Milagros Rangel, Neetha Basheer, Patricia Place, Romina Romero, Steven Tam, Trung Nguyen, Abey Thomas, Alexander Alex Frolov, Alka Khera, Amy Browning, Brendan Kelley, Courtney Dawson, Dana Mathews, Elaine Most, Elizeva Ellie Phillips, Lynn Nguyen, Maribel Nunez, Matalin Miller, Matthew R. Jones, Natalie Martinez, Rebecca Logan, Roderick McColl, Sari Pham, Tiffani Fox, Tracey Moore, Allan Levey, Abby Brown, Andrea Kippels, Ashton Ellison, Casie Lyons, Chadwick Hales, Cindy Parry, Courtney Williams, Elizabeth McCorkle, Guy Harris, Heather Rose, Inara Jooma, Jahmila Al-Amin, James Lah, James Webster, Jessica Swiniarski, Latasha Chapman, Laura Donnelly, Lauren Mariotti, Mary Locke, Phyllis Vaughn, Rachael Penn, Sallie Carpentier, Samira Yeboah, Sarah Basadre, Sarah Malakauskas, Stefka Lyron, Tara Villinger, Terra Burney, Jeffrey Burns, Ala Abusalim, Alexandra Dahlgren, Alexandria Montero, Anne Arthur, Heather Dooly, Katelynn Kreszyn, Katherine Berner, Lindsey Gillen, Maria Scanlan, Mercedes Madison, Nicole Mathis, Phyllis Switzer, Ryan Townley, Samantha Fikru, Samantha Sullivan, Ella Wright, Maryam Beigi, Anthony Daley, Ashley Ko, Brittney Luong, Glen Nyborg, Jessica Morales, Kelly Durbin, Lauren Garcia, Leila Parand, Lorena Macias, Lorena Monserratt, Maya Farchi, Pauline Wu, Robert Hernandez, Thao Rodriguez, Neill Graff-Radford, A‘llana Marolt, Anton Thomas, Deborah Aloszka, Ercilia Moncayo, Erin Westerhold, Gregory Day, Kandise Chrestensen, Mary Imhansiemhonehi, Sanna McKinzie, Sochenda Stephens, Sylvia Grant, Jared Brosch, Amy Perkins, Aubree Saunders, Debra Silberberg Kovac, Heather Polson, Isabell Mwaura, Kassandra Mejia, Katherine Britt, Kathy King, Kayla Nichols, Kayley Lawrence, Lisa Rankin, Martin Farlow, Patricia Wiesenauer, Robert Bryant, Scott Herring, Sheryl Lynch, Skylar Wilson, Traci Day, William Korst, Christopher van Dyck, Adam Mecca, Alyssa Miller, Amanda Brennan, Amber Khan, Audrey Ruan, Carol Gunnoud, Chelsea Mendonca, Danielle Raynes-Goldfinger, Elaheh Salardini, Elisa Hidalgo, Emma Cooper, Erawadi Singh, Erin Murphy, Jeanine May, Jesse Stanhope, Jessica Lam, Julia Waszak, Kimberly Nelsen, Kimberly Sacaza, Mayer Joshua Hasbani, Meghan Donahue, Ming-Kai Chen, Nicole Barcelos, Paul Eigenberger, Robin Bonomi, Ryan O‘Dell, Sarah Jefferson, Siddharth Khasnavis, Stephen Smilowitz, Susan DeStefano, Susan Good, Terry Camarro, Vanessa Clayton, Yanis Cavrel, YuQuan ″Oliver″ Lu, Howard Chertkow, Howard Bergman, Chris Hosein, Sandra Black, Anish Kapadia, Aparna Bhan, Benjamin Lam, Christopher Scott, Gillian Gabriel, Jennifer Bray, Ljubica Zotovic, Maria Samira Gutierrez, Mario Masellis, Marjan Farshadi, Maurylette Gui, Meghan Mitchell, Rebecca Taylor, Ruby Endre, Zhala Taghi-Zada, Robin Hsiung, Carolyn English, Ellen Kim, Eugene Yau, Haley Tong, Laura Barlow, Lauren Jennings, Michele Assaly, Paula Nunes, Tahlee Marian, Andrew Kertesz, John Rogers, Dick Trost, Dylan Wint, Charles Bernick, Donna Munic, Ian Grant, Aaliyah Korkoyah, Ali Raja, Allison Lapins, Caila Ryan, Jelena Pejic, Kailey Basham, Leena Lukose, Loreece Haddad, Lucas Quinlan, Nathaniel Houghtaling, Carl Sadowsky, Walter Martinez, Teresa Villena, Brigid Reynolds, Angelica Forero, Carolyn Ward, Emma Brennan, Esteban Figueroa, Giuseppe Esposito, Jessica Mallory, Kathleen Johnson, Kathryn Turner, Katie Seidenberg, Kelly McCann, Margaret Bassett, Melanie Chadwick, Raymond Scott Turner, Robin Bean, Saurabh Sharma, Gad Marshall, Aferdita Haviari, Alison Pietras, Bradley Wallace, Catherine Munro, Gladiliz Rivera-Delpin, Hadley Hustead, Isabella Levesque, Jennifer Ramirez, Karen Nolan, Kirsten Glennon, Mariana Palou, Michael Erkkinen, Nicole DaSilva, Pamela Friedman, Regina M. Silver, Ricardo Salazar, Roxxanne Polleys, Scott McGinnis, Seth Gale, Tia Hall, Tuan Luu, Steven Chao, Emmeline Lin, Jaila Coleman, Kevin Epperson, Minal Vasanawala, Alireza Atri, Amy Rangel, Brittani Evans, Candy Monarrez, Carol Cline, Carolyn Liebsack, Daniel Bandy, Danielle Goldfarb, Debbie Intorcia, Jennifer Olgin, Kelly Clark, Kelsey King, Kylee York, Marina Reade, Michael Callan, Michael Glass, Michaela Johnson, Michele Gutierrez, Molly Goddard, Nadira Trncic, Parichita Choudhury, Priscilla Reyes, Serena Lowery, Shaundra Hall, Sonia Olgin, Stephanie de Santiago, Michael Alosco, Alyssa Ton, Amanda Jimenez, Andrew Ellison, Brandon Anderson, Della Carter, Donna Veronelli, Steven Lenio, Eric Steinberg, Jesse Mez, Jason Weller, Jennifer Johns, Jessica Harkins, Alexa Puleio, Ina Hoti, Jane Mwicigi, Olivia Schultz, Mona Lauture, Ridiane Denis, Ronald Killiany, Sarab Singh, Wendy Qiu, Ycar Devis, Thomas Obisesan, Andrew Stone, Debra Ordor, Ifreke Udodong, Immaculata Okonkwo, Javed Khan, Jillian Turner, Kyliah Hughes, Oshoze Kadiri, Charles Duffy, Ariana Moss, Katherine Stapleton, Maria Toth, Marianne Sanders, Martin Ayres, Melissa Hamski, Parianne Fatica, Paula Ogrocki, Sarah Ash, Stacy Pot, Doris Chen, Andres Soto, Costin Tanase, David Bissig, Hafsanoor Vanya, Heather Russell, Hitesh Patel, Hongzheng Zhang, Kelly Wallace, Kristi Ayers, Maria Gallegos, Martha Forloines, Meghan Sinn, Queennie Majorie S. Kahulugan, Richard Isip, Sandra Calderon, Talia Hamm, Michael Borrie, T-Y Lee, Rob Bartha, Sterling Johnson, Sanjay Asthana, Cynthia M. Carlsson, Allison Perrin, Pierre Tariot, Adam Fleisher, Stephanie Reeder, Horacio Capote, Allison Emborsky, Anna Mattle, Bela Ajtai, Benjamin Wagner, Bennett Myers, Daryn Slazyk, Delaney Fragale, Erin Fransen, Heather Macnamara, Jonathan Falletta, Joseph Hirtreiter, Laszlo Mechtler, Megan King, Michael Asbach, Michelle Rainka, Richard Zawislak, Scott Wisniewski, Stephanie O’Malley, Tatiana Jimenez-Knight, Todd Peehler, Traci Aladeen, Vernice Bates, Violet Wenner, Wisam Elmalik, Douglas W. Scharre, Arun Ramamurthy, Soumya Bouchachi, Maria Kataki, Rawan Tarawneh, Brendan Kelley, Dzintra Celmins, Alicia Leader, Chris Figueroa, Heather Bauerle, Katlynn Patterson, Michael Reposa, Steven Presto, Tuba Ahmed, Wendy Stewart, Godfrey D. Pearlson, Karen Blank, Karen Anderson, Robert B. Santulli, Eben S. Schwartz, Jeff Williamson, Alicia Jessup, Andrea Williams, Crystal Duncan, Abigail O’Connell, Karen Gagnon, Ezequiel Zamora, James Bateman, Freda Crawford, Deb Thompson, Eboni Walker, Jennifer Rowell, Mikell White, Phillip “Hunter” Ledford, Sarah Bohlman, Susan Henkle, Joseph Bottoms, Lena Moretz, Bevan Hoover, Michael Shannon, Samantha Rogers, Wendy Baker, William Harrison, Chuang-Kuo Wu, Alexis DeMarco, Ava Stipanovich, Daniel Clark, Jennifer Davis, Kerstin Doyon, Marie Amoyaw, Mauro Veras Acosta, Ronald Bailey, Scott Warren, Terry Fogerty, Victoria Sanborn, Meghan Riddle, Stephen Salloway, Paul Malloy, Stephen Correia, Charles Windon, Morgan Blackburn, Howard J. Rosen, Bruce L. Miller, Amanda Smith, Ijeoma Mba, Jenny Echevarria, Juris Janavs, Emily Roglaski, Meagan Yong, Rebecca Devine, Hamid Okhravi, Edgardo Rivera, Teresa Kalowsky, Caroline Smith, Christina Rosario, Joseph Masdeu, Richard Le, Maushami Gurung, Marwan Sabbagh, Angelica Garcia, Micah Ellis Slaughter, Nadeen Elayan, Skieff Acothley, Nunzio Pomara, Raymundo Hernando, Vita Pomara, Chelsea Reichert, Olga Brawman-Mintzer, Allison Acree, Arthur Williams, Campbell Long, Rebecca Long, Paul Newhouse, Sydni Jenee Hill, Amy Boegel, Sudha Seshadri, Amy Saklad, Floyd Jones, William Hu, V. Sotelo, Yaneicy Gonazalez Rojas, Jacobo Mintzer, Crystal Flynn Longmire, Kenneth Spicer

**Affiliations:** 1 Department of Genetics and Genomic Sciences, Icahn School of Medicine at Mount Sinai, One Gustave L. Levy Place , New York, NY 10029 USA; 2 Mount Sinai Center for Transformative Disease Modeling, Icahn School of Medicine at Mount Sinai, One Gustave L. Levy Place , New York, NY 10029 USA; 3 Icahn Institute for Data Science and Genomic Technology, Icahn School of Medicine at Mount Sinai, One Gustave L. Levy Place, New York, NY 10029 USA; 4 Department of Artificial Intelligence and Human Health, Icahn School of Medicine at Mount Sinai, One Gustave L. Levy Place, New York, NY 10029 USA; 5 Nash Family Department of Neuroscience, Icahn School of Medicine at Mount Sinai, 1425 Madison Avenue, New York, NY 10029 USA; 6 Department of Psychiatry, Icahn School of Medicine at Mount Sinai, 1425 Madison Avenue, New York, NY 10029 USA; 7 Icahn School of Medicine at Mount Sinai, Alzheimer’s Disease Research Center, New York, NY 10029 USA; 8 Psychiatry, JJ Peters VA Medical Center, 130 West Kingsbridge Road, Bronx, NY 10468 USA; 9 N. Bud Grossman Center for Memory Research and Care, Department of Neurology, University of Minnesota, Minneapolis, MN 55455 USA; 10 Geriatric Research Education & Clinical Center (GRECC), Minneapolis VA Health Care System, Minneapolis, MN 55417 USA

**Keywords:** Alzheimer’s disease, Sex, Apolipoprotein E, Diffusion tensor imaging, gene coexpression network analysis

## Abstract

Alzheimer’s disease (AD) disproportionately affects women and carriers of the apolipoprotein E ε4 allele (APOE4), yet little is known about how sex and APOE interact to influence white matter (WM) integrity during disease progression. We integrated diffusion MRI and matched blood transcriptomic data to investigate these interactions and their underlying biological mechanisms. WM microstructure was quantified using diffusion tensor imaging (DTI) and neurite orientation dispersion and density imaging (NODDI), and regional vulnerability was assessed with a composite vulnerability score (CVS) derived from associations between diffusion features and AD severity across clinical traits in each of the four sex-APOE groups (female or male, with or without APOE4). Brain parcellation with the Eve atlas revealed regions consistently affected across sex-APOE groups (e.g., parahippocampal and superior temporal gyri) and regions specific to individual groups (e.g., the cingulum in females with APOE4 and the middle frontal gyrus in males without APOE4). Gene co-expression network analysis of the matched blood expression data identified gene subnetworks linked to group-specific regional vulnerability, including a muscle tissue morphogenesis module regulated by *NEURL1B* and *HIST1H2BN* associated with middle frontal gyrus vulnerability. These findings demonstrate that sex and APOE genotype jointly shape region-specific WM vulnerability and its molecular signatures in AD. Understanding these interactions provides novel mechanistic insights and may inform precision approaches to drug development, biomarker discovery, and clinical trial design for AD.

**Supplementary Information:**

The online version contains supplementary material available at https://doi.org/10.1007/s11357-025-02089-4.

## Introduction

Alzheimer’s disease (AD) is a heterogeneous, multifactorial disease with considerable clinicopathological variability. Sex is a major risk factor that shapes both lifetime risk and disease phenotype. In the USA, more than two-thirds of individuals with AD at age 65 and older are women, whose estimated lifetime risk of developing AD is nearly twice that of men. Women also tend to exhibit more severe neuropathology, greater brain atrophy, and faster cognitive decline [1–3]. These sex-related differences are further modified by apolipoprotein E (APOE) genotype, the strongest genetic risk factor for late-onset AD (LOAD) [4]. Women aged 65–75 with APOE ε3/ε4 have increased AD risk, while those with APOE ε2/ε3 show reduced risk compared to men [5].

Previous research has examined sex × APOE interactions in relation to AD biomarkers (tau and Aβ in CSF and PET), brain atrophy, functional connectivity, and cognition [6–10]. However, their impact on white matter (WM) microstructure, a key substrate of AD pathophysiology, remains underexplored. WM degeneration occurs early in AD and may precede evident cortical atrophy [11]. Diffusion MRI provides sensitive markers of WM integrity, and studies have shown that both sex and APOE4 influence diffusion-based metrics. For example, women often exhibit reduced fractional anisotropy (FA) and increased radial diffusivity (RD) in major WM tracts, such as the cingulum in healthy individuals, and in the anterior thalamic radiation (ATR) and corticospinal tract (CST) in AD patients [12–14]. Similarly, APOE4 carriers (APOE ε2/ε4, ε3/ε4, or ε4/ε4) show more microstructural disruptions in the cingulum, corpus callosum, and internal capsule compared to noncarriers (APOE ε2/ε3 or ε3/ε3) [15]. More recent work further suggests that among healthy elderly, APOE4-related WM changes may be more pronounced in women, particularly in fibers such as the cingulum, entorhinal region, and corona radiata [16, 17]. These findings highlight the need to systematically investigate sex- and APOE-specific WM vulnerability during AD progression.

Our group has previously identified sex- and APOE-specific expressed genes in AD using large-scale brain transcriptomic data, and demonstrated that key molecular drivers, such as LRP10, contribute to sex- and APOE genotype-dependent AD pathology [18]. We also identified the hippocampus as the WM region most vulnerable in AD, with the CELF1 locus SNP rs2203712 showing the strongest association with hippocampal diffusion features in an integrative imaging–genetic framework [19]. Together, this work highlights how systems biology approaches that integrate neuroimaging, transcriptomic, and clinical data can advance understanding of the mechanisms underlying AD heterogeneity.

In the present study, participants were stratified into four groups based on biological sex and APOE statuses (i.e., Female-APOE4, Female-nonAPOE4, Male-APOE4, and Male-nonAPOE4). APOE4 groups included carriers of one or more ε4 alleles (ε2/ε4, ε3/ε4, and ε4/ε4 genotypes), consistent with epidemiological evidence showing that APOE4 increases AD risk with odds ratios ranging from 2.6 to 14.9 [20]. We developed a composite vulnerability score (CVS) to quantify associations between regional WM microstructural integrity and clinical markers of AD severity, and integrated these findings with matched blood gene expression data. This design allowed us to uncover the molecular networks driving sex- and APOE-specific WM vulnerability, thereby advancing understanding of AD pathogenesis and informing precision strategies for diagnosis, therapy, and clinical trial design.

## Materials and methods

### Study participants

Data were obtained from the Alzheimer’s Disease Neuroimaging Initiative (ADNI; adni.loni.usc.edu). Baseline diffusion-weighted imaging (DWI) scans were acquired for each participant. Diagnostic categories included cognitively normal (CN), significant memory concerns (SMC), early mild cognitive impairment (EMCI), late MCI (LMCI), MCI, and AD. For this study, we included EMCI, LMCI, MCI, and AD participants (CDR = 0.5–1.0) to capture the full continuum of AD development. Our CVS approach is designed to leverage this disease spectrum by quantifying associations between regional WM integrity and clinical severity across multiple stages. This strategy enhances statistical power by maximizing the dynamic range of diffusion MRI and clinical measures, identifies regional vulnerability patterns that may be missed in stage-restricted analyses, and reflects the heterogeneity observed in real-world clinical settings.

Participants were further stratified into four sex-APOE groups: females with APOE4 (Female-APOE4), females without APOE4 (Female-nonAPOE4), males with APOE4 (Male-APOE4), and males without APOE4 (Male-nonAPOE4). APOE4 carriers included individuals with ε2/ε4, ε3/ε4, or ε4/ε4 genotypes, while noncarriers included ε2/ε2, ε2/ε3, or ε3/ε3 (Supplementary Table 1). Analyses of regional WM integrity were conducted in 235 participants with available sex, APOE genotype, and 13 clinical traits (48 Female-APOE4, 47 Female-nonAPOE4, 72 Male-APOE4, and 68 Male-nonAPOE4; Table 1, Supplementary Tables 2, 3). Among these, 125 participants also had matched blood transcriptomic data, enabling the identification of gene subnetworks, enriched pathways, and key driver genes associated with regional vulnerability in each group (24 Female-APOE4, 30 Female-nonAPOE4, 29 Male-APOE4, and 42 Male-nonAPOE4; Table 1, Supplementary Table 4).

**Table 1:** Demographic and clinical characteristics of ADNI participants in sex-APOE groups

Groups	Female-APOE4	Female-nonAPOE4	Male-APOE4	Male-nonAPOE4
Neuroimaging + APOE genotype + clinical traits				
***N***	48	47	72	68
**Age (SD)** **Race (white)**	71.06 (7.51) 47	75.69 (9.03) 40	73.63 (7.50) 67	75.04 (6.82) 65
Neuroimaging + APOE genotype + clinical traits + gene expression				
***N***	24	30	29	42
**Age (SD)** **Race (white)**	70.45 (7.70) 23	77.30(8.84) 24	74.06 (6.88) 25	74.55 (6.75) 40

### Data acquisitions and processing

Typical DWI acquisition parameters were as follows: field strength = 3.0 T; manufacturer = GE or Siemens Medical Systems; flip angle = 90°; gradient directions = 41; matrix size = 128 × 128 or 232 × 232; voxel size = 1.3 × 1.3 × 2.7 mm or 2 × 2 × 2 mm; TE = 57 ms; TR = 9050 or 7200 ms.

Data preprocessing was performed in FSL 6.0.5, which included correction for eddy currents, head motion, and susceptibility-induced distortions. Brain extraction was carried out using BET with parameters optimized for diffusion-weighted images. Basic bias field correction was applied to address intensity inhomogeneities across the imaging volume. Quality control was conducted through visual inspection of all preprocessing outputs. Diffusion properties were estimated using diffusion tensor imaging (DTI) and neurite orientation dispersion and density imaging (NODDI), which provides a more precise characterization of microstructural substrates [21]. Eight DTI features were derived using tensor fitting and “FSLMATHS,” including FA, RD, mean diffusivity (MD), axial diffusivity (AxD), linearity of the tensor (LIN), mode of the tensor (MOD), planarity of the tensor (PLA), and sphericity of the tensor (SPH) [22]. The NODDI model generated the orientation dispersion index (ODI) using MATLAB R2022a, providing insights into neurite orientation dispersion [23] (further details on the algorithm and clinical significance are provided in the Supplementary Materials).

After computing nine DWI features for each subject, diffusion space was registered to a common space by projecting the FA map onto a standard template, the FA image of the JHU-MNI “Eve” atlas [24] (mristudio.org). Registration was performed using linear (“FLIRT”) and non-linear (“FNIRT”) transformations. The transformation matrix was applied to the remaining DWI features, and segmentation-based analysis used the “Eve” atlas white matter parcellation map (WMPM) Type I to parcellate the brain into 176 regions, enabling voxel-wise statistics for the nine DWI features.

### Quantification of regional vulnerability

**Fig. 1:**
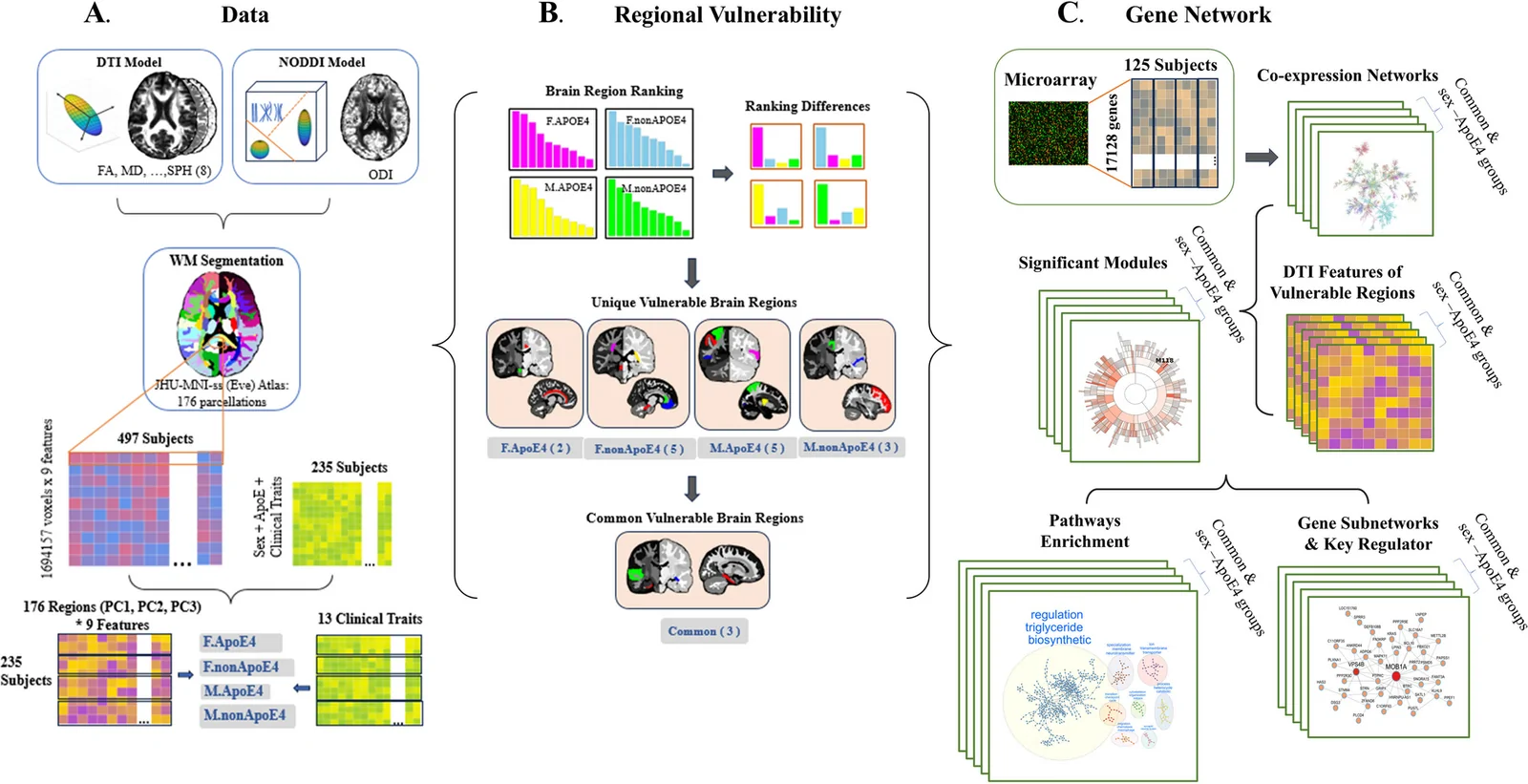
The analytical framework for identifying brain regional vulnerability and associated gene subnetworks across sex-APOE groups. **A** The diffusion MRI data from the ADNI cohort were processed using diffusion tensor imaging (DTI) and neurite orientation dispersion and density imaging (NODDI) models. White matter (WM) was segmented into 176 regions using the Eve atlas, and voxel-based diffusion metrics were extracted. Principal component analysis was then applied to reduce dimensionality, yielding three principal components (PCs) per feature for each region. **B** Regional vulnerability was assessed by ranking the strength of associations between clinical traits and neuroimaging features (DTI- and NODDI-derived PCs) in each brain region in each sex-SPOE group. This approach identified both regions consistently vulnerable across all sex-APOE groups and regions uniquely vulnerable in each group. **C** Gene co-expression networks were constructed for all participants and those in each sex-APOE group. The relevance of each gene module to AD was determined by correlating module eigengenes with diffusion MRI features of brain regions in the corresponding group

Following DWI feature analysis, voxel-level data for each brain region was reduced with principal component analysis (PCA) using “prcomp” in R [25]. The top three principal components (PCs) were retained for each of the nine DWI features, resulting in a total of 27 PCs per region (Fig. 1A). Thirteen neuropsychological assessments were collected at baseline to measure AD severity, and they are Clinical Dementia Rating Sum of Boxes (CDRSB), AD Assessment Scale (ADAS), Mini-Mental State Examination (MMSE), Montreal Cognitive Assessment (MoCA), Functional Activities Questionnaire (FAQ), Rey Auditory Verbal Learning Test (RAVLT), and patient and study partner recorded Measurement of Everyday Cognitive (ECog) (Supplementary Table 3).

To evaluate the impact of different brain regions on AD severity, we performed separate linear regression models for each brain region (denoted as ${R}_{i}$, *i* = 1 to 176) on each neuropsychological trait (denoted as ${T}_{j}$, *j* = 1 to 13). The regression model is formulated as follows:

1 $${T}_{ij}={\beta }_{0}^{(ij)}+{\sum }_{m=1}^{9}{\sum }_{n=1}^{3}{\beta }_{mn}^{(ij)}{PC}_{imn}$$

where

- ${T}_{ij}$denotes the impact of region ${R}_{i}$ on trait ${T}_{j}$.
- ${\beta }_{0}^{(ij)}$is the intercept specific to ${R}_{i}$ and ${T}_{j}.$
- ${\beta }_{mn}^{(ij)}$are the coefficients capturing the impact of the *n*-th PC of the *m*-th DWI feature of ${R}_{i}$ on ${T}_{j}.$
- ${PC}_{imn}$refers to the PCs from ${R}_{i}$, where *m* indexes the nine DWI features and *n* indexes the three PCs for each feature.

The double summation captures the impact of all nine DWI features and their three PCs within each region on neuropsychological trait ${T}_{j}$. A higher F-statistic and a lower *p*-value indicate a stronger predictive power of a brain region’s DWI features in explaining trait variation associated with AD severity.

To comprehensively evaluate the impact of a specific brain region on AD severity and to quantitatively rank the vulnerability of each region for comparison across groups, we computed the composite vulnerability score (CVS). The CVS was calculated by summing the negative natural logarithms of *p*-values for each region across traits and then normalizing these scores across regions:

2 $${CVS}_{{R}_{i}}=\frac{{\sum }_{j=1}^{13}(-\mathrm{ln} \left({P}_{F}^{\left(i,j\right)}\right))}{\mathrm{max}({\sum }_{j=1}^{13}(-\text{ln }\left({P}_{F}^{\left(i,j\right)}\right)))}$$

where

- ${CVS}_{{R}_{i}}$represents the CVS for region ${R}_{i}.$
- ${P}_{F}^{(i,j)}$denotes the *p*-value of the F-statistic from the regression of all DWI features in ${R}_{i}$ on ${T}_{j}$, as defined in Eq. (1).

The CVS for each brain region was calculated by summing its predictive power across 13 neuropsychological traits and normalizing by the highest score among 176 regions, yielding a score between 0 and 1, with 1 representing a region with the highest vulnerability. Regions were ranked by CVS independently in each sex-APOE group. Common vulnerable regions were identified by examining the overlap among the top 30 most vulnerable brain regions across the four sex-APOE groups, whereas group-specific vulnerable regions were defined as those ranked in the top 10% for one group but in the bottom 60% for the others (Fig. 1B). We excluded the brain regions that appeared in different groups, irrespective of their brain hemispheres.

### Correlation between gene co-expression network and regional vulnerability

We integrated the regional DWI data with the gene expression profiles from 125 matched ADNI subjects to identify system-level biological processes associated with the most vulnerable white matter region unique to each sex-APOE group. Gene expression from blood samples, processed using the Affymetrix Human Genome U219 array, underwent normalization with robust multi-array analysis (RMA) and log2 transformation. Probe sets with missing or multiple genes were removed, and those with the highest variance were retained. Low-expression genes (lowest 10%) were excluded, leaving 17,128 genes used for co-expression network analysis for all the subjects and each sex-APOE group.

Based on the preprocessed gene expression data, gene co-expression networks were built for all subjects and each sex-APOE group using Multiscale Embedded Gene Co-expression Network Analysis (MEGENA) in R [26]. Pearson correlation coefficients (PCCs) were calculated for all the gene pairs and then selected the gene pairs with significant PCC (i.e., false discovery rate (FDR) < 0.05). The gene pairs showing significant correlations were ranked based on correlation strength and subsequently utilized to build a Planar Filtered Network (PFN) through the Planar Maximally Filtered Graph (PMFG) algorithm. We then applied multiscale clustering analysis (MCA) to identify gene co-expression modules at various scales of compactness. To assess the statistical significance of those modules, we compared them to random PFN modules generated by shuffling the link weights of the parent cluster. Additionally, we conducted a multiscale hub analysis (MHA) to identify highly connected hub genes within each significant module. Modules containing fewer than ten genes were excluded from further analysis (Fig. 1C**)**.

To identify gene modules associated with regional susceptibility, we computed module eigengenes, defined as the first PC of each module’s gene expression profile. These eigengenes were then correlated with the top three PCs derived from nine DWI features within each sex-APOE group. For each region–module pair, we quantified aggregated significance (*p* < 0.05) using a Fisher-based metric (Fisher Sum) calculated as the sum of the $-{\mathrm{log}}_{10}(p)$ values across all contributing tests. The significance of a module to a selected vulnerable WM region was ranked by Fisher Sum.

The sex-APOE4 group–specific signaling pathway map was generated by extracting the Gene Ontology (GO) [27] biological process hierarchy network for pathways significantly enriched (adjusted *p* < 0.05) in the top five gene modules most strongly associated with DWI features of the vulnerable brain regions. Human gene–GO annotations and ontology structure were retrieved from the org.Hs.eg.db [28] and GO.db [29] packages in R. Module-specific GO biological process enrichment was assessed using a hypergeometric test, in which each gene module was evaluated for GO term overrepresentation relative to the full gene universe (*p* < 0.05), and the resulting biological process terms were Benjamini–Hochberg–adjusted and extracted for downstream pathway clustering. All significant GO terms were then grouped according to their biological process categories to summarize system-level functional themes. The resulting sex-APOE4–specific signaling maps were visualized using Cytoscape (Version 3.10.4).

## Results

### Shared regional vulnerabilities

**Fig. 2:**
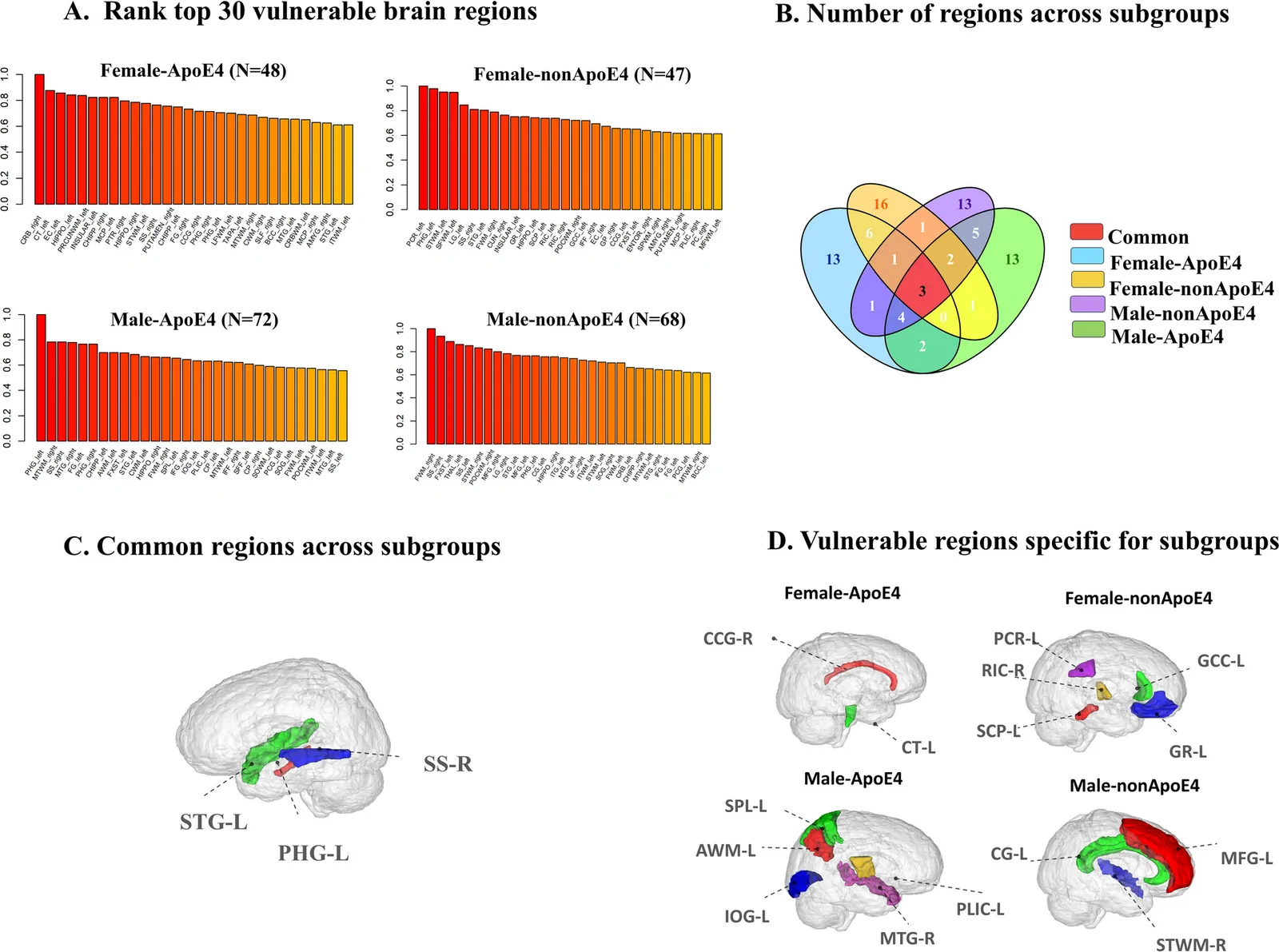
AD-vulnerable brain regions shared by sex-APOE groups or specific to each patient group. **A** Top 30 brain regions ranked by composite vulnerability scores (CVS) for each sex-APOE group. Higher CVS values indicate greater vulnerability to AD (see Supplementary Fig. 1 for enhanced visualization). **B** Venn diagram showing the overlap and uniqueness of AD-vulnerable regions across the four groups, based on the top 30 regions from panel A. **C** Brain regions consistently identified as vulnerable across all four groups, as determined from the overlap analysis in panel B. **D** Brain regions uniquely vulnerable within each sex-APOE group. Abbreviations: CT-L, corticospinal tract (left); CCG-R, cingulum (cingulate gyrus, right); AWM-L, angular white matter (left); SPL-L, superior parietal lobule (left); IOG-L, inferior occipital gyrus (left); PLIC-L, posterior limb of internal capsule (left); MTG-R, middle temporal gyrus (right); SCP-L, superior cerebellar peduncle (left); GCC-L, genu of corpus callosum (left); GR-L, gyrus rectus (left); RIC-R, retrolenticular part of the internal capsule (right); PCR-L, posterior corona radiata (left); MFG-L, middle frontal gyrus (left); CG-L, cingulate gyrus (left); STWM-R, superior temporal white matter (right); PHG-L, parahippocampal gyrus (left); STG-L, superior temporal gyrus (left); SS-R, sagittal stratum (right)

To assess regional vulnerability, we analyzed the associations between nine DWI features (and their three PCs) and clinical traits across the four sex-APOE groups: Female-APOE4 (*N* = 48), Female-nonAPOE4 (*N* = 47), Male-APOE4 (*N* = 72), and Male-nonAPOE4 (*N* = 68) (Fig. 2A; Supplementary Fig. 1).

Commonly vulnerable regions were identified by examining the top 30 most vulnerable regions in each group (Fig. 2B). Three regions consistently were ranked among the most vulnerable across groups: the left parahippocampal gyrus (PHG-L), left superior temporal gyrus (STG-L), and right sagittal stratum (SS-R) (Fig. 2C). These findings were consistent with our prior transcriptomic analysis showing the PHG as highly vulnerable at the molecular level [30] and with a diffusion MRI study ranking SS-R, PHG-L, and STG-L among the top 50 vulnerable regions in a population comprising CN, SMC, EMCI, LMCI, and AD groups [19].

### Group-specific regional vulnerabilities

We next examined unique regional vulnerabilities within each sex-APOE group, defined as regions in the top 10% (≥ 90th percentile) of vulnerability for one group but at the bottom 60% (≤ 60th percentile) for the other three (Table 2; Fig. 2D**)**.

**Table 2:** Regional vulnerability specific to each sex-APOE group**.** The values in the table represent the percentile rank of the selected regions among a total of 176 regions investigated for each sex-APOE group. For example, a value of 90 represents the 90th percentile, indicating that the region ranks in the top 10% among the regions investigated

Region	Female-APOE4	Female-nonAPOE4	Male-APOE4	Male-nonAPOE4
Female-APOE4				
**CT-L**	99	10	22	19
**CCG-R**	91	28	36	23
Female-nonAPOE4				
**SCP-L**	31	93	5	20
**GCC-L**	44	91	15	2
**GR-L**	14	94	56	5
**RIC-R**	44	92	47	1
**PCR-L**	60	100	38	60
Male-APOE4				
**AWM-L**	13	9	96	21
**SPL-L**	1	55	93	15
**IOG-L**	47	3	91	34
**PLIC-L**	51	14	91	30
**MTG-R**	34	11	98	51
Male-nonAPOE4				
**MFG-L**	12	47	52	94
**CG-L**	60	52	19	93
**STWM-R**	55	57	38	97

*CT-L* left: corticospinal tract, *CCG-R:* right cingulum (cingulate gyrus), *SCP-L:* left superior cerebellar peduncle, *GCC-L:* left genu of corpus callosum, *GR-L:* left gyrus rectus, *RIC-R:* right retrolenticular part of internal capsule, *PCR-L:* left posterior corona radiata, *AWM-L:* left angular white matter, *SPL-L:* left superior parietal lobule, *IOG-L:* left inferior occipital gyrus, *PLIC-L:* left posterior limb of internal, *MTG-R:* right middle temporal gyrus, *MFG-L:* left middle frontal gyrus, *CG-L:* left cingulate gyrus, *STWM-R:* right superior temporal white matter

Brain regions were excluded from selection if the same regions were present in different sex-APOE groups, regardless of the hemisphere

In the Female-APOE4 group, the left corticospinal tract (CT-L) and right cingulum (CCG-R) were uniquely vulnerable. In the Female-nonAPOE4 group, unique vulnerabilities included the left superior cerebellar peduncle (SCP-L), left genu of the corpus callosum (GCC-L), left gyrus rectus (GR-L), right retrolenticular internal capsule (RIC-R), and left posterior corona radiata (PCR-L). In the Male-APOE4 group, the left angular white matter (AWM-L), left superior parietal lobule (SPL-L), left inferior occipital gyrus (IOG-L), left posterior limb of the internal capsule (PLIC-L), and right middle temporal gyrus (MTG-R) were uniquely vulnerable. Finally, in the Male-nonAPOE4 group, the left middle frontal gyrus (MFG-L), left cingulate gyrus (CG-L), and right superior temporal white matter (STWM-R) were unique vulnerabilities.

Together, these results suggested that although some regions were commonly affected, others displayed sex- and APOE-specific WM vulnerabilities, highlighting the heterogeneity of AD progression.

### Cognitive measures linked to vulnerability

To determine which neuropsychological traits contributed most to the identification of sex-APOE–specific vulnerable regions, we examined correlations between regional diffusion MRI features and multiple clinical measures of AD severity (see the “Materials and methods” section for the details of computing CVS).

**Fig. 3:**
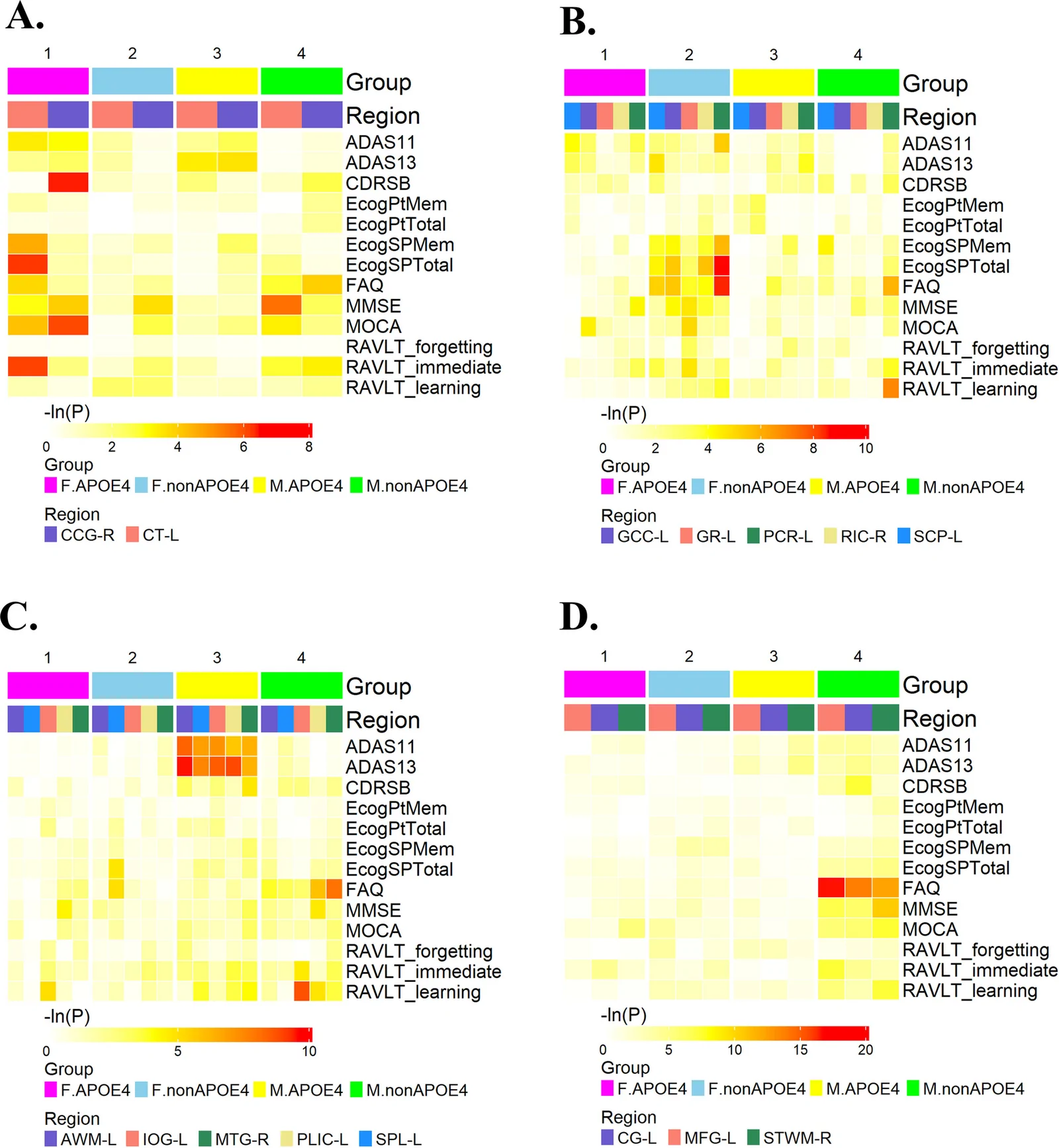
Associations between regional diffusion MRI features and clinical traits across sex-APOE groups. Heatmaps display correlations between diffusion features of specific brain regions and 13 clinical traits in each sex-APOE group. Color intensity reflects the negative natural logarithm of the regression *p*-value; higher values indicate stronger associations between regional DWI features and clinical traits. **A** In the Female-APOE4 group, the right cingulum (CCG-R) and left corticospinal tract (CT-L) showed the strongest correlations, distinct from other groups. **B** In the Female-nonAPOE4 group, the left genu of the corpus callosum (GCC-L), left gyrus rectus (GR-L), left posterior corona radiata (PCR-L), right retrolenticular internal capsule (RIC-R), and left superior cerebellar peduncle (SCP-L) were most strongly associated with clinical traits. **C** In the Male-APOE4 group, the left angular white matter (AWM-L), left inferior occipital gyrus (IOG-L), right middle temporal gyrus (MTG-R), left posterior limb of the internal capsule (PLIC-L), and left superior parietal lobule (SPL-L) showed strong correlations. **D** In the Male-nonAPOE4 group, the left cingulate gyrus (CG-L), left middle frontal gyrus (MFG-L), and right superior temporal white matter (STWM-R) were significantly associated with clinical traits

In Fig. 3, the Female-APOE4 group showed significantly stronger correlations between diffusion features of the CCG-R and CT-L and clinical traits compared with the other groups, particularly with MMSE and MoCA, which captured global cognitive performance and assessed a broad spectrum of cognitive functions [31, 32]. In the Female-nonAPOE4 group, diffusion features of the SCP-L, GCC-L, GR-L, RIC-R, and PCR-L were most strongly associated with clinical traits, correlating most consistently with ECog and FAQ, measures of everyday cognitive function and daily activities [33, 34].

In the Male-APOE4 group, diffusion features of the AWM-L, SPL-L, IOG-L, PLIC-L, and MTG-R demonstrated robust correlations with clinical traits, especially ADAS, a comprehensive scale that evaluated both cognitive and non-cognitive symptoms, such as mood and behavior, associated with AD [31]. In the Male-nonAPOE4 group, diffusion features of the MFG-L, CG-L, and STWM-R were significantly associated with clinical traits, with the strongest correlations observed with FAQ.

### Gene subnetworks associated with regional vulnerability in sex-APOE groups

**Fig. 4:**
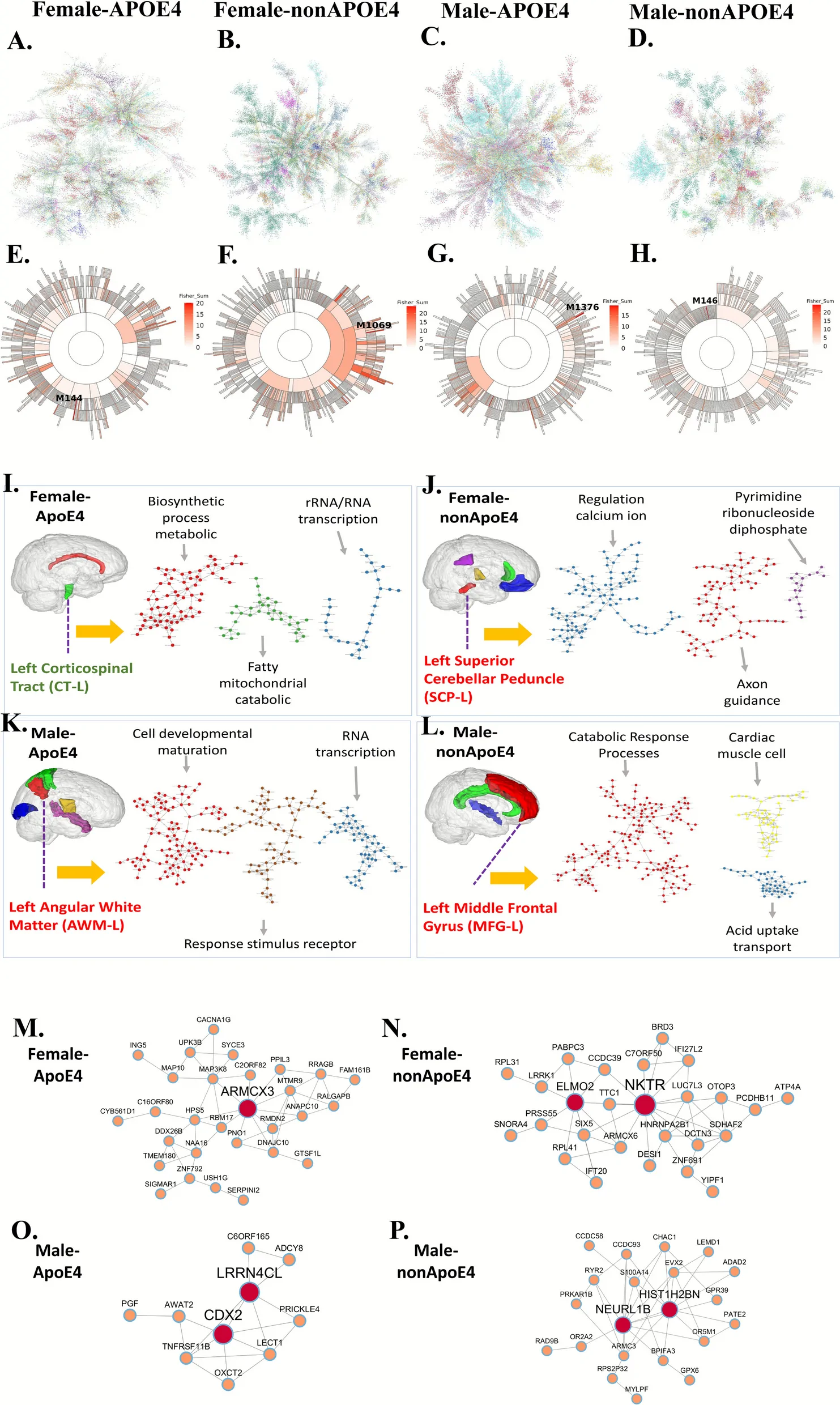
Gene co-expression networks and functional enrichment analyses across sex-APOE groups. MEGENA co-expression networks constructed for each sex-APOE group: Female-APOE4 (**A**), Female-nonAPOE4 (**B**), Male-APOE4 (**C**), and Male-nonAPOE4 (**D**). Sunburst plots summarize the module hierarchies and Fisher-sum significant associations (*p* < 0.05) between gene modules and diffusion features for the most vulnerable white matter regions in each group—CT-L in Female-APOE4 (**E**), SCP-L in Female-nonAPOE4 (**F**), AWM-L in Male-APOE4 (**G**), and MFG-L in Male-nonAPOE4 (**H**). Each block represents a module, with color intensity indicating the Fisher-sum statistic reflecting the combined significance of correlations between module eigengenes and regional DWI features. The sex-APOE–specific signaling pathway maps based on the Gene Ontology Biological Process (GO-BP) hierarchy for Female-APOE4 (**I**), Female-nonAPOE4 (**J**), Male-APOE4 (**K**), and Male-nonAPOE4 (**L**) were generated from GO-BP terms significantly enriched (adjusted *p* < 0.05) in the top five modules most significantly associated with the most vulnerable white matter regions described above. For clarity, only the largest three functional clusters are shown in the figure; the complete visualization of all clusters is provided in Supplementary Fig. 3. Each node represents an enriched GO-BP term, while the colored clusters denote higher-level functional groups obtained by summarizing GO-BP terms with similar biological themes; clusters containing fewer than ten GO terms were excluded. The top gene modules/subnetworks showing the strongest Fisher-sum significance with the most vulnerable regions in Female-APOE4 (**M**), Female-nonAPOE4 (**N**), Male-APOE4 (**O**), and Male-nonAPOE4 (**P**) (as labeled in Fig. 4B), along with their key regulatory genes. Red nodes represent hub genes, and node size reflects each gene’s connectivity within the subnetwork (larger nodes indicate higher connectivity). Gene co-expression networks and functional enrichment analyses for all participants are shown in Supplementary Fig. 2 for additional detail

We conducted gene co-expression network analysis on matched blood transcriptomic data from 125 participants (24 Female-APOE4, 30 Female-nonAPOE4, 29 Male-APOE4, 42 Male-nonAPOE4) to identify molecular pathways associated with sex-APOE–specific regional vulnerabilities. The analysis generated 546 co-expression modules across all participants, 790 in the Female-APOE4 group, 866 in the Female-nonAPOE4 group, 815 in the Male-APOE4 group, and 704 in the Male-nonAPOE4 group (Fig. 4A–D;Supplementary Fig. 2A). Each network was constructed independently; therefore, while modules were labeled using the same naming convention (e.g., M1, M2, …), modules with the same name in different networks represent distinct sets of co-expressed genes. Modules can range from small subnetworks containing a few dozen genes to larger clusters comprising several hundred genes, reflecting different levels of biological organization.

To identify the molecular signatures underlying sex-APOE4–specific WM vulnerability, we focused on the most vulnerable region in each subgroup, as these regions captured the earliest and most robust biological signatures of subgroup-specific pathology. Accordingly, we selected PHG-L for all participants, CT-L in Female-APOE4, SCP-L in Female-nonAPOE4, AWM-L in Male-APOE4, and MFG-L in Male-nonAPOE4. Fisher Sum (see the “Materials and methods” section for the details of computing Fisher Sum) was then calculated to aggregate significance across correlations between each gene module and the full set of diffusion-weighted imaging features.

### Shared molecular signatures

Among all the modules, M118 collectively impacted PHG-L the most, having the highest Fisher Sum score, followed by M332, M855, M245, and M171 (Supplementary Fig. 2B; Supplementary Table 5). We investigated significantly enriched biological pathways (adjusted *p* < 0.05) within these top modules, identifying a signaling network centered on triglyceride biosynthetic regulation (the largest functional cluster), followed by membrane neurotransmitter transport, ion transmembrane transport, heterocycle catabolic processes, cell cycle transition, macrophage migration, cytoskeleton mitosis, and synaptic vesicle fusion (Supplementary Fig. 2C).

These pathways revealed convergent immune–metabolic and signaling vulnerabilities shared across sex-APOE4 groups. The largest cluster, triglyceride biosynthesis, reflected a peripheral lipid–immune state linked to AD pathology [35–37]. Within the cluster, GO terms like response to stimulus, immune system process, and cytokine regulation indicated stimulus-driven metabolic reprogramming in circulating immune cells, while tolerance induction, nerve impulse transmission, and membrane potential regulation reflected lipid-sensitive neuroimmune signaling. Additional enrichment of neurotransmitter transport, ion transport, and synaptic vesicle fusion highlighted communication programs in leukocytes. Heterocycle catabolic indicated metabolic stress during immune activation, while cell cycle transition and cytoskeleton–mitosis reflected proliferative and structural remodeling responses, and macrophage migration signaled increased chemotactic activity.

Given its strongest association with PHG-L vulnerability, we further examined M118. This module was enriched for dephosphorylation and regulated by the hub genes MOB1A and VPS4B (Supplementary Fig. 2D). Protein dephosphorylation fell within the cell cycle transition-labeled cluster and represented a critical regulatory mechanism in cell cycle control relevant to aging and AD. Notably, the phosphorylation–dephosphorylation balance in peripheral blood profoundly affected brain tau hyperphosphorylation, for example through PP2A dysfunction [38]. Both hub genes demonstrated AD-relevant functions: MOB1A, a scaffolding protein in the Hippo signaling pathway, regulated cell proliferation through phosphorylation-dependent mechanisms [39], while VPS4B, a component of the ESCRT machinery essential for endosomal trafficking and autophagy, showed genetic variants associated with late-onset AD risk [40].

### Group-specific molecular signatures

In the Female-APOE4 group, M144 impacted CT-L the most, followed by M809, M1774, M1096, and M310 (Fig. 4E; Supplementary Table 6). We identified a signaling network map centered on biosynthetic process metabolism, fatty mitochondrial catabolism, and rRNA/RNA transcription as the most prominent clusters (Fig. 4I). Additional enriched pathways included transport of intracellular lipid, mitochondrion cilium, membrane node cell, and response to stimulus radiation (Supplementary Fig. 3A).

These pathways suggested immunometabolic and mitochondrial–transcriptional dysregulation signatures in female APOE4 carriers. These concurrent pathway enrichments may reflect altered metabolic flux as circulating immune cells attempted to balance anabolic demands with energy production. Recent work demonstrated that APOE4 females showed accelerated endocrine aging, with failure of adaptive bioenergetic reprogramming, decreased mitochondrial function, and increased neuroinflammation/demyelination [41]. A large metabolomics study further showed that APOE4 + females had greater deficits in mitochondrial energy production metabolites compared with other groups [42]. The enrichment of rRNA/RNA transcription pathways reflected increased ribosome biogenesis and protein synthesis demands, suggesting that despite mitochondrial impairment, these cells may maintain metabolically costly transcriptional programs that could contribute to accelerated immune aging in female APOE4 carriers.

We further examined M144, enriched for response to nickel cation and regulated by the hub gene ARMCX3 (Fig. 4M). The enrichment of nickel response pathways fell within the response stimulus radiation–labeled cluster and may reflect broader metal ion homeostasis and stress response mechanisms [43]. ARMCX3, an X-linked mitochondrial protein and member of the ALEX/ARMCX mitochondrial trafficking protein family, supported mitochondrial transport, dynamics, and neuronal survival [44], and its ancestral gene Armc10 protected against Aβ-induced toxicity [45].

In the Female-nonAPOE4 group, M1069 impacted SCP-L the most, followed by M1507, M1682, M1146, and M465 (Fig. 4F; Supplementary Table 7). We identified a signaling network map centered on regulation of calcium ion, axon guidance, and pyrimidine ribonucleoside diphosphate as the most prominent clusters (Fig. 4J). Additional enriched pathways included spermidine biosynthesis, morphogenesis of tract septum, alanine metabolism, and icosanoid secretion of lipid (Supplementary Fig. 3B).

These pathways suggested coordinated immune–metabolic integration signatures in female nonAPOE4 carriers. Ca^2^⁺-dependent signaling was essential for the activation and function of lymphocytes within the peripheral immune system [46]. Axon-guidance molecules—including semaphorins—were increasingly recognized as key regulators of immune-cell migration and trafficking [47]. Enrichment of pyrimidine and UDP/CDP metabolic pathways was consistent with cellular states of elevated biosynthetic demand, as pyrimidine nucleotides were indispensable for T-cell DNA synthesis, proliferation, and survival [48].

We further examined M1069, enriched for protein–FAD linkage and regulated by the hub genes NKTR and ELMO2 (Fig. 4N). The enrichment of FAD (flavin adenine dinucleotide)–related terms fell within the alanine metabolism–labeled cluster. FAD was a major mitochondrial redox coenzyme required for the activity of flavoproteins involved in oxidative metabolism [49]. NKTR was best characterized as a regulator of cytoskeletal remodeling and activation in NK cells and T cells. ELMO2 regulated cytoskeletal reorganization necessary for cell migration and engulfment of apoptotic cells [50].

In the Male-APOE4 group, M1376 impacted AWM-L the most, followed by M158, M76, M591, and M846 (Fig. 4G; Supplementary Table 8). We identified a signaling network map centered on cell developmental maturation, response stimulus receptor, and RNA transcription as the most prominent clusters (Fig. 4K). Additional enriched pathways included regulation of secretion exocytosis, regulation of polymerization of microtubule, dephosphorylation, and myoblast proliferation (Supplementary Fig. 3C).

These pathways suggested systemic immuno-developmental and transcriptional reprogramming signatures in male APOE4 carriers. Enrichment of cell developmental maturation reflected broad reactivation of developmental and morphogenetic gene programs, signatures that typically indicated stress-induced activation of progenitor-like or differentiation pathways under conditions of systemic inflammatory pressure or disrupted hematopoietic regulation [51]. APOE4 was increasingly recognized as a systemic immune modulator, with APOE4 carriers displaying conserved, disease-independent immune-related proteomic alterations across plasma, CSF, and brain [52]. Concurrent enrichment of response stimulus receptor and RNA transcription aligned with APOE4-associated dysregulation of immune-cell responsiveness and global transcriptional programs.

We further examined M1376, enriched for fatty acid derivative metabolic process and regulated by the hub genes LRRN4CL and CDX2 (Fig. 4O). The enrichment of M1376 fell within the cell developmental maturation–labeled cluster, as fatty acid oxidation and related lipid pathways were key regulators of T-cell activation and survival in vivo [53]. CDX2 was a homeobox transcription factor known to reconfigure hematopoietic gene programs [54], whereas LRRN4CL remained poorly characterized.

In the Male-nonAPOE4 group, M146 impacted MFG-L the most, followed by M327, M324, M121, and M570 (Fig. 4H; Supplementary Table 9). We identified a signaling network map centered on catabolic response processes, cardiac muscle cells, and acid uptake transport as the most prominent clusters (Fig. 4L). Additional enriched pathways included myeloid differentiation macrophages, ion activity, sister mitotic activity, blood pressure, miRNA silencing, microtubule polymerization depolymerization, and lymphocyte chemotaxis (Supplementary Fig. 3D).

These pathways suggested systemic metabolic and cytoskeletal reprogramming signatures in male nonAPOE4 carriers. The enrichment of catabolic response reflected activation of metabolic stress–adaptive programs widely used by circulating immune cells during oxidative challenge and inflammation, consistent with AMPK- and catabolism-linked immune regulation [55]. Although annotated under cardiac muscle cell cluster due to GO hierarchical structure, this cluster reflected actin–myosin and calcium-signaling pathways widely used by immune cells, as they are essential for T-cell activation [56, 57]. Enrichment of acid transport reflected amino acid, organic acid, and nucleotide-sugar transport, as circulating immune cells depended on sustained uptake of glutamate, arginine, and other nutrients to support metabolic reprogramming [58].

We further examined M146, enriched for muscle tissue morphogenesis and regulated by the hub genes NEURL1B and HIST1H2BN (Fig. 4P). The enrichment of M146 fell within the cardiac muscle–labeled cluster. NEURL1B, a member of the E3 ubiquitin ligases family, participated in Notch signaling pathways that regulated cellular differentiation [59], while HIST1H2BN, a histone H2B variant, was associated with chromatin remodeling during cellular reprogramming [60].

Taken collectively, these results suggested that although immune-related pathways were shared correlates of common WM vulnerabilities, the sex- and APOE-specific molecular signatures revealed distinct immune–metabolic–transcriptional profiles across subgroups, highlighting the biological heterogeneity that characterizes AD progression.

## Discussion

We systematically investigated the influence of sex and APOE genotypes on WM integrity across early symptomatic and clinical stages of AD. By integrating voxel-wise DTI and NODDI features with neuropsychological traits, we identified regional WM vulnerabilities that were unique to each sex-APOE group or shared across the four sex-APOE groups. Importantly, integrating these imaging based findings with blood-derived gene co-expression networks uncovered potential molecular signatures of WM abnormalities, highlighting the value of multimodal approaches for understanding AD heterogeneity.

Across the sex-APOE groups, WM vulnerability was observed in the PHG-L, STG-L, and SS-R. These regions are aligned with Braak’s early staging of AD pathology, where tau aggregates began in the entorhinal/transentorhinal regions and extended to temporal areas [61]. Prior DTI studies have consistently reported reduced FA in the PHG and STG among individuals with MCI and AD [62, 63], reflecting compromised WM connectivity in pathways supporting memory, auditory processing, and language. The involvement of the SS, a projection pathway connecting the thalamus and occipital lobe, further highlighted the widespread WM vulnerability during AD progression and was consistent with a previous study reporting increased diffusivity in AD patients compared with controls [64].

We also observed sex- and APOE-specific vulnerabilities in tracts such as the cingulum, corticospinal tract, and corpus callosum across the AD continuum. These findings provided new evidence that sex and APOE4 status interacted to modulate WM integrity, not only in healthy aging [16, 17], but also during AD progression. In group-specific analyses, females tended to show more motor- and limbic-related vulnerabilities, whereas males exhibited greater involvement of cognitive and sensory pathways. Notably, APOE4 status appeared to induce more selective vulnerabilities in females (affecting primarily motor and limbic tracts) but more distributed vulnerabilities in males (spanning parietal–temporal, visual, and cognitive regions). Across groups, most vulnerabilities were left-lateralized, suggesting possible hemispheric asymmetry in WM susceptibility. However, these patterns need be replicated in larger studies to confirm the generalizability of sex-APOE4 interactions.

At the molecular level, shared immune–metabolic activation was observed across all four subgroups, indicating that circulating immune cells engage common stress-responsive and energetically demanding programs. These findings underscore the central role of neuroinflammation in AD, with peripheral immune alterations potentially mirroring or contributing to central white matter disruption [35, 65]. In group-specific analyses, APOE4 status was associated with more bioenergetically constrained and metabolically intensive dysregulation in females, but a more transcriptionally and developmentally reprogrammed profile in males. In contrast, nonAPOE4 groups exhibited more controlled, sex-specific immune–metabolic strategies. Sex differences shifted the dominant mode of immune dysregulation, with females primarily exhibiting metabolic and mitochondrial alterations and males showing broader developmental and cytoskeletal adaptations. These findings highlight distinct biological processes that may influence white matter vulnerability in a sex- and genotype-dependent manner.

The group-specific WM and molecular vulnerability patterns identified in this study have important implicationsfor advancing precision medicine in aging and AD. First, the presence of sex- and APOE4-dependent WM susceptibility highlights the need to establish sex- and APOE4-specific diagnostic thresholds for neuroimaging biomarkers. Developing sex- and APOE4-stratified normative reference values for WM integrity may improve early detection for individuals at greater risk. The group differences observed in WM–clinical associations further reinforce this need: Female-APOE4 carriers show the strongest associations between WM vulnerability and global cognition (MMSE, MoCA), whereas Male-APOE4 carriers have the strongest associations with broader cognitive and behavioral impairment (ADAS). These group-specific clinical signatures are aligned with the evidence that sex-specific cognitive norms improve diagnostic accuracy [66], suggesting that both cognitive and neuroimaging biomarkers require sex- and APOE4-aware interpretation. Second, the differential mitochondrial, biosynthetic, developmental, and catabolic programs across groups support sex- and APOE4-tailored prevention and treatment strategies, consistent with the findings that APOE4 modifies responsiveness to hormone therapy in females [67], and estrogen’s neuroprotective effects depend on intact mitochondrial and calcium regulation [68]. Third, incorporating these group-specific neuroimaging and molecular signatures into clinical trial design—through sex- and APOE4-stratified enrollment or analysis—may reduce biological heterogeneity and enhance therapeutic efficacy, as recent anti-amyloid trials indicated sex-dependent treatment response [69, 70].

This study has several limitations. The retrospective nature using the ADNI data prevented matching covariates like age, race, education, and medical history to groups by sex and APOE status, limiting causal conclusions. Although ADNI remained the largest and most suitable cohort with matched DWI and transcriptomic data, only a moderate number of subjects were included for DWI, and fewer for transcriptomic analysis, reducing the statistical power to draw robust conclusions. Additionally, the study sample predominantly comprised white participants, limiting the generalizability of the findings to non-white populations, given that the prevalence and effects of APOE4 vary across racial groups [71]. Finally, a more detailed comparison between APOE4 dosage (no copies, one copy, or two copies of the APOE4 allele) and WM integrity was lacking due to the limited sample size.

In conclusion, our results demonstrated that WM microstructural changes during AD progression were not uniform but were influenced by sex and genetic background. Integrating peripheral gene expression with neuroimaging provided new opportunities to link network biology with brain aging and may help identify biomarkers for early detection and personalized intervention. Future work should extend these analyses to longitudinal data to clarify how sex-APOE interactions modulate trajectories of WM decline and to determine whether targeted modulation of immune, metabolic, or transcriptional pathways can mitigate AD-related vulnerabilities across different subpopulations.

## Supplementary Information

Below is the link to the electronic supplementary material.

ESM 1 (5. 39 MB DOCX)

ESM 2 (647 KB XLSX)

## Data Availability

Data used in preparation of this article were obtained from the Alzheimer’s Disease Neuroimaging Initiative (ADNI) database (adni.loni.usc.edu). As such, the investigators within the ADNI contributed to the design and implementation of ADNI and/or provided data but did not participate in analysis or writing of this report. A complete listing of ADNI investigators can be found at https://adni.loni.usc.edu/wp-content/uploads/how_to_apply/ADNI_Acknowledgement_List.pdf.
