# Supplementary material for: Sex and APOE genotype specific brain regional vulnerability to Alzheimer's Disease
Source: GeroScience. 2026 Feb 18;48(6):8591–608. doi: 10.1007/s11357-025-02089-4 (PMC13638851; doi:10.1007/s11357-025-02089-4)
Supplement: Supplementary file 1 [file 11357_2025_2089_MOESM1_ESM.docx]

**Supplementary Information for “****Sex- and APOE Genotype–Specific Regional White Matter Vulnerability to Alzheimer’s Disease”**

**Main Text Abbreviation**

| AD | Alzheimer's Disease | MCI | Mild Cognitive Impairment |
| --- | --- | --- | --- |
| ADAS | Alzheimer's Disease Assessment Scale | MEGENA | Multiscale Embedded Gene Co-Expression Network Analysis |
| ADNI | Alzheimer's Disease Neuroimaging Initiative | MHA | Multiscale Hub Analysis |
| ANCOVA | Analysis of Covariance | MMSE | Mini-Mental State Examination |
| AxD | Axial Diffusivity | MOCA | Montreal Cognitive Assessment |
| CDRSB | Clinical Dementia Rating Sum of Boxes | MOD | Mode of the Tensor |
| CN | Cognitive Normal | NODDI | Neurite Orientation Dispersion and Density Imaging |
| CNS | Central Nervous System | OASIS | Open Access Series of Imaging Studies |
| CSF | Cerebrospinal Fluid | ODI | Orientation Dispersion Index |
| CVS | Composite Vulnerability Score | PC | Principal Component |
| DTI | Diffusion Tensor Imaging | PCA | Principal Component Analysis |
| DWI | Diffusion Weighted Imaging | PFN | Planar Filtered Network |
| ECOG | Everyday Cognitive | PLA | Planarity of the Tensor |
| FA | Fractional Anisotropy | RAVLT | Rey Auditory Verbal Learning Test |
| FAQ | Functional Activities Questionnaire | RD | Radial Diffusivity |
| FDR | False Discovery Rate | SMC | Significant Memory Concerns |
| GM | Grey Matter | SPH | Sphericity of the Tensor |
| HSV | Herpes Simplex Virus | VPS | Vacuolar Protein-Sorting Protein |
| LIN | Linearity of the Tensor | WAM | White Matter-Associated Microglia |
| LOAD | Late Onset Alzheimer's Disease | WM | White Matter |
| MD | Mean Diffusivity |  |  |

**Abbreviations for 176 White Matter Structures**

| SPL-L | Superior Parietal Lobule Left | SPL-R | Superior Parietal Lobule Right |
| --- | --- | --- | --- |
| CG-L | Cingulate Gyrus Left | CG-R | Cingulate Gyrus Right |
| SFG-L | Superior Frontal Gyrus Left | SFG-R | Superior Frontal Gyrus Right |
| MFG-L | Middle Frontal Gyrus Left | MFG-R | Middle Frontal Gyrus Right |
| IFG-L | Inferior Frontal Gyrus Left | IFG-R | Inferior Frontal Gyrus Right |
| PCG-L | Precentral Gyrus Left | PCG-R | Precentral Gyrus Right |
| POCG-L | Postcentral Gyrus Left | POCG-R | Postcentral Gyrus Right |
| AG-L | Angular Gyrus Left | AG-R | Angular Gyrus Right |
| PC-L | Pre-Cuneus Left | PC-R | Pre-Cuneus Right |
| CUN-L | Cuneus Left | CUN-R | Cuneus Right |
| LG-L | Lingual Gyrus Left | LG-R | Lingual Gyrus Right |
| FG-L | Fusiform Gyrus Left | FG-R | Fusiform Gyrus Right |
| PHG-L | Parahippocampal Gyrus Left | PHG-R | Parahippocampal Gyrus Right |
| SOG-L | Superior Occipital Gyrus Left | SOG-R | Superior Occipital Gyrus Right |
| IOG-L | Inferior Occipital Gyrus Left | IOG-R | Inferior Occipital Gyrus Right |
| MOG-L | Middle Occipital Gyrus Left | MOG-R | Middle Occipital Gyrus Right |
| ENTOR-L | Entorhinal Area Left | ENTOR-R | Entorhinal Area Right |
| STG-L | Superior Temporal Gyrus Left | STG-R | Superior Temporal Gyrus Right |
| ITG-L | Inferior Temporal Gyrus Left | ITG-R | Inferior Temporal Gyrus Right |
| MTG-L | Middle Temporal Gyrus Left | MTG-R | Middle Temporal Gyrus Right |
| LFG-L | Lateral Fronto Orbital Gyrus Left | LFG-R | Lateral Fronto Orbital Gyrus Right |
| MFOG-L | Middle Fronto Orbital Gyrus Left | MFOG-R | Middle Fronto Orbital Gyrus Right |
| SG-L | Supramarginal Gyrus Left | SG-R | Supramarginal Gyrus Right |
| GR-L | Gyrus Rectus Left | GR-R | Gyrus Rectus Right |
| INSULAR-L | Insular Left | INSULAR-R | Insular Right |
| AMYG-L | Amygdala Left | AMYG-R | Amygdala Right |
| HIPPO-L | Hippocampus Left | HIPPO-R | Hippocampus Right |
| CRB-L | Cerebellum Left | CRB-R | Cerebellum Right |
| CT-L | Corticospinal Tract Left | CT-R | Corticospinal Tract Right |
| ICP-L | Inferior Cerebellar Peduncle Left | ICP-R | Inferior Cerebellar Peduncle Right |
| ML-L | Medial Lemniscus Left | ML-R | Medial Lemniscus Right |
| SCP-L | Superior Cerebellar Peduncle Left | SCP-R | Superior Cerebellar Peduncle Right |
| CP-L | Cerebral Peduncle Left | CP-R | Cerebral Peduncle Right |
| ALIC-L | Anterior Limb of Internal Capsule Left | ALIC-R | Anterior Limb of Internal Capsule Right |
| PLIC-L | Posterior Limb of Internal Capsule Left | PLIC-R | Posterior Limb of Internal Capsule Right |
| PTR-L | Posterior Thalamic Radiation Left | PTR-R | Posterior Thalamic Radiation Right |
| ACR-L | Anterior Corona Radiata Left | ACR-R | Anterior Corona Radiata Right |
| SCR-L | Superior Corona Radiata Left | SCR-R | Superior Corona Radiata Right |
| PCR-L | Posterior Corona Radiata Left | PCR-R | Posterior Corona Radiata Right |
| CCG-L | Cingulum (Cingulate Gyrus) Left | CCG-R | Cingulum (Cingulate Gyrus) Right |
| CHIPP-L | Cingulum (Hippocampus) Left | CHIPP-R | Cingulum (Hippocampus) Right |
| FXST-L | Fornix(Cres) Stria Terminalis Left | FXST-R | Fornix(Cres) Stria Terminalis Right |
| SLF-L | Superior Longitudinal Fasciculus Left | SLF-R | Superior Longitudinal Fasciculus Right |
| SFF-L | Superior Fronto Occipital Fasciculus Left | SFF-R | Superior Fronto Occipital Fasciculus Right |
| IFF-L | Inferior Fronto Occipital Fasciculus Left | IFF-R | Inferior Fronto Occipital Fasciculus Right |
| SS-L | Sagittal Stratum Left | SS-R | Sagittal Stratum Right |
| EC-L | External Capsule Left | EC-R | External Capsule Right |
| UF-L | Uncinate Fasciculus Left | UF-R | Uncinate Fasciculus Right |
| PCT-L | Pontine Crossing Tract Left | PCT-R | Pontine Crossing Tract Right |
| MCP-L | Middle Cerebellar Peduncle Left | MCP-R | Middle Cerebellar Peduncle Right |
| FCB-L | Fornix (Column And Body) Left | FX-R | Fornix Right |
| GCC-L | Genu of Corpus Callosum Left | GCC-R | Genu of Corpus Callosum Right |
| BCC-L | Body of Corpus Callosum Left | BCC-R | Body of Corpus Callosum Right |
| SCC-L | Splenium of Corpus Callosum Left | SCC-R | Splenium of Corpus Callosum Right |
| RIC-L | Retrolenticular Part of Internal Capsule Left | RIC-R | Retrolenticular Part of Internal Capsule Right |
| RN-L | Red Nucleus Left | RN-R | Red Nucleus Right |
| SN-L | Substantia Nigra Left | SN-R | Substantia Nigra Right |
| TAPA-L | Tapetum Left | TAPA-R | Tapetum Right |
| CN-L | Caudate Nucleus Left | CN-R | Caudate Nucleus Right |
| PUTAMEN-L | Putamen Left | PUTAMEN-R | Putamen Right |
| THAL-L | Thalamus Left | THAL-R | Thalamus Right |
| GP-L | Globus Pallidus Left | GP-R | Globus Pallidus Right |
| MIDBRAIN-L | Midbrain Left | MIDBRAIN-R | Midbrain Right |
| PONS-L | Pons Left | PONS-R | Pons Right |
| MEDULLA-L | Medulla Left | MEDULLA-R | Medulla Right |
| SPWM-L | Superior Parietal WM Left | SPWM-R | Superior Parietal WM Right |
| CWM-L | Cingulum WM Left | CWM-R | Cingulum WM Right |
| SFWM-L | Superior Frontal WM Left | SFWM-R | Superior Frontal WM Right |
| MFWM-L | Middle Frontal WM Left | MFWM-R | Middle Frontal WM Right |
| IFWM-L | Inferior Frontal WM Left | IFWM-R | Inferior Frontal WM Right |
| PRCWM-L | Precentral WM Left | PRCWM-R | Precentral WM Right |
| POCWM-L | Postcentral WM Left | POCWM-R | Postcentral WM Right |
| AWM-L | Angular WM Left | AWM-R | Angular WM Right |
| PRCUNWM-L | Pre Cuneus WM Left | PRCUNWM-R | Pre Cuneus WM Right |
| CUNWM-L | Cuneus WM Left | CUNWM-R | Cuneus WM Right |
| LWM-L | Lingual WM Left | LWM-R | Lingual WM Right |
| FWM-L | Fusiform WM Left | FWM-R | Fusiform WM Right |
| SOWM-L | Superior Occipital WM Left | SOWM-R | Superior Occipital WM Right |
| IOWM-L | Inferior Occipital WM Left | IOWM-R | Inferior Occipital WM Right |
| MOWM-L | Middle Occipital WM Left | MOWM-R | Middle Occipital WM Right |
| STWM-L | Superior Temporal WM Left | STWM-R | Superior Temporal WM Right |
| ITWM-L | Inferior Temporal WM Left | ITWM-R | Inferior Temporal WM Right |
| MTWM-L | Middle Temporal WM Left | MTWM-R | Middle Temporal WM Right |
| LFWM-L | Lateral Fronto Orbital WM Left | LFWM-R | Lateral Fronto Orbital WM Right |
| MFOWM-L | Middle Fronto Orbital WM Left | MFOWM-R | Middle Fronto Orbital WM Right |
| SWM-L | Supramarginal WM Left | SWM-R | Supramarginal WM Right |
| RWM-L | Rectus WM Left | RWM-R | Rectus WM Right |
| CRBWM-L | Cerebellum WM Left | CRBWM-R | Cerebellum WM Right |

**Supplementary Table 1. Detailed breakdown of APOE genotypes across sex-APOE groups.** APOE4 carriers include individuals with APOE ε2/ε4, ε3/ε4, and ε4/ε4 genotypes. NonAPOE4 carriers include those with APOE ε2/ε2, ε2/ε3, and ε3/ε3 genotypes.

| Groups | Female-ApoE4 | Female-nonApoE4 | Male-ApoE4 | Male-nonApoE4 |
| --- | --- | --- | --- | --- |
| APOE ε4/ε4 | 9 | 0 | 15 | 0 |
| APOE ε3/ε4 | 37 | 0 | 55 | 0 |
| APOE ε2/ε4 | 2 | 0 | 2 | 0 |
| APOE ε3/ε3 | 0 | 42 | 0 | 57 |
| APOE ε2/ε3 | 0 | 4 | 0 | 11 |
| APOE ε2/ε2 | 0 | 1 | 0 | 0 |

**Supplementary Table 2. The number of individuals in each diagnosis group with the availability of both APOE genotype and clinical traits to identify the most vulnerable brain regions for each sex-ApoE group.**

| APOE allele status by gender | EMCI | LMCI | MCI | AD |
| --- | --- | --- | --- | --- |
| Female-ApoE4 | 12 | 16 | 6 | 14 |
| Male-ApoE4 | 33 | 20 | 2 | 17 |
| Female-nonApoE4 | 28 | 7 | 6 | 6 |
| Male-nonApoE4 | 38 | 12 | 6 | 12 |

**Supplementary Table 3.** **The number of participants and the characteristic of clinical traits in the sex-APOE groups for neuroimaging & clinical regression analysis.**

| Groups | Female-APOE4 | Female-nonAPOE4 | Male-APOE4 | Male-nonAPOE4 |
| --- | --- | --- | --- | --- |
| CDRSB | 48 | 47 | 72 | 68 |
| Mean (SD) | 2.33 (1.86) | 1.80 (1.77) | 2.18 (1.44) | 1.76 (1.72) |
| ADAS11 | 48 | 47 | 72 | 68 |
| Mean (SD) | 12.66 (6.71) | 9.89 (6.30) | 12.56 (6.35) | 10.72 (7.65) |
| ADAS13 | 47 | 47 | 72 | 68 |
| Mean (SD) | 19.57 (9.46) | 16.08 (8.83) | 19.94 (8.77) | 16.57 (10.20) |
| MMSE | 48 | 47 | 72 | 68 |
| Mean (SD) | 26.44 (2.95) | 27.23 (2.42) | 26.75 (2.52) | 27.50 (2.54) |
| RAVLT_immediate | 48 | 47 | 72 | 68 |
| Mean (SD) | 33.31 (12.27) | 37.09 (11.81) | 29.72 (8.63) | 32.24 (11.71) |
| RAVLT_learning | 48 | 47 | 72 | 68 |
| Mean (SD) | 3.96 (2.75) | 4.60 (2.55) | 3.61 (2.35) | 4.31 (2.29) |
| RAVLT_forgetting | 48 | 47 | 72 | 68 |
| Mean (SD) | 5.40 (2.90) | 4.15 (2.58) | 4.62 (1.92) | 4.37 (2.37) |
| FAQ | 48 | 47 | 72 | 68 |
| Mean (SD) | 5.52 (6.67) | 3.83 (7.18) | 5.68 (6.33) | 4.51 (6.84) |
| MOCA | 45 | 44 | 72 | 64 |
| Mean (SD) | 21.58 (4.43) | 22.23 (4.01) | 21.90 (3.34) | 22.14 (4.23) |
| EcogPtMem | 46 | 44 | 72 | 66 |
| Mean (SD) | 2.43 (0.79) | 2.38 (0.87) | 2.40 (0.75) | 2.11 (0.66) |
| EcogPtTotal | 45 | 44 | 72 | 66 |
| Mean (SD) | 1.78 (0.59) | 1.89 (0.71) | 1.86 (0.60) | 1.63 (0.41) |
| EcogSPMem | 46 | 44 | 72 | 66 |
| Mean (SD) | 2.52 (0.95) | 2.16 (0.91) | 2.53 (0.85) | 2.22 (0.82) |
| EcogSPTotal | 46 | 44 | 71 | 66 |
| Mean (SD) | 2.00 (0.83) | 1.74 (0.76) | 2.10 (0.79) | 1.79 (0.65) |

**Supplementary Table 4. The number of individuals in each diagnosis group with the availability of both APOE genotype, clinical traits and gene expression data to identify genetic networks and key drivers associated with the vulnerable brain regions in each sex-ApoE group.**

| APOE allele status by gender | EMCI | LMCI | MCI | AD |
| --- | --- | --- | --- | --- |
| Female-ApoE4 | 8 | 9 | 2 | 5 |
| Male-ApoE4 | 16 | 6 | 0 | 7 |
| Female-nonApoE4 | 24 | 2 | 3 | 1 |
| Male-nonApoE4 | 29 | 9 | 2 | 2 |

**Supplementary Figure 1.** **Enhanced visualization of Figure 2(A), providing a clearer, more detailed view of the top 30 brain regions ranked by composite vulnerability scores (CVS) for each sex-APOE group.** Regions with higher CVS values are more susceptible to AD pathology.

**Supplementary Figure 2. Gene co-expression networks and functional enrichment analyses for all participants.** A) MEGENA co-expression networks. (B) Module hierarchies and Fisher-sum associations with the top vulnerable region (PHG-L). (C) Summary of enriched GO Biological Process terms from the top five significant modules. (D) Key subnetworks and hub genes with the strongest Fisher-sum associations.

**Supplementary Figure 3. Completed visualization of Figure 4 (I–L), providing a clearer and more detailed view of the completed pathways for each sex–APOE group following the methods section.**

**B.**

**A.**

**D.**

**C.**

**Supplementary algorithms and their clinical significance**

As stated in the main text, the DTI and NODDI features are derived using the following algorithms:

FA =$\sqrt{\frac{3}{2}}\frac{\sqrt{{(\lambda1-\lambda)}^{2}+({\lambda2-\lambda)}^{2}+{(\lambda3-\lambda)}^{2}}}{\sqrt{{{{\lambda1}^{2}+\lambda2}^{2}+\lambda3}^{2}}}$

MD = $\lambda$ = $\frac{\lambda1+ \lambda2+\lambda3}{3}$

AD = λ1

RD =$\frac{\lambda2+\lambda3}{2}$

LIN = $\frac{\lambda1- \lambda2}{\lambda1+ \lambda2+\lambda3}$

MOD = $\frac{\lambda1\lambda2\lambda3}{{\sqrt{{(\lambda1-\lambda)}^{2}+({\lambda2-\lambda)}^{2}+{(\lambda3-\lambda)}^{2}}}^{3}}$

PLA =$\frac{2(\lambda2- \lambda3)}{\lambda1+ \lambda2+\lambda3}$

SPH = $\frac{3\lambda3}{\lambda1+ \lambda2+\lambda3}$

ODI = $\frac{2}{\pi}$ arctan($\frac{1}{\kappa}$)

Where λ1, λ2, and λ3 are the eigenvalues of the diffusion tensor and κ is the estimated concentration parameter describing the dispersion of neurite orientations.

Specifically, FA (fractional anisotropy) quantifies the degree of anisotropy of water diffusion within tissues. It ranges from 0 to 1, where 0 represents isotropic diffusion (equal in all directions, as in cerebrospinal fluid) and 1 represents highly anisotropic diffusion (preferential in one direction, as in white matter tracts). Higher FA values generally indicate healthy white matter integrity, whereas lower values may suggest damage or degeneration of fiber tracts. MD (mean diffusivity) measures the overall magnitude of water diffusion, calculated as the average of the diffusion along three principal axes of the diffusion tensor. It reflects the ease with which water molecules diffuse through tissue. Higher MD values can indicate microstructural damage, edema, or increased extracellular space, as seen in neurodegenerative diseases. RD (radial diffusivity) reflects the magnitude of water diffusion perpendicular to the main direction of fiber tracts. Higher RD values are often associated with demyelination, as water can diffuse more freely when the myelin sheath surrounding axons is damaged. AxD (axial diffusivity) measures diffusion along the principal direction of fibers. A decrease in AD might suggest axonal damage, which restricts diffusion along the length of the fiber. LIN (linearity of the tensor) assesses the linearity of the diffusion tensor, with higher values indicating the presence of elongated structures like WM fiber bundles. MOD (mode of the tensor) categorizes anisotropy into three types, where values close to -1 indicate diffusion along a geometric plane, values close to +1 indicate diffusion along a single direction, and values close to 0 indicate isotropic diffusion. PLA (planarity of the tensor) measures the extent to which the diffusion tensor deviates from a perfect cylindrical shape, with values closer to 1 indicating greater deviation. SPH (sphericity of the tensor) indicates how closely the diffusion tensor resembles a spherical shape, with values closer to 0 suggesting more isotropic diffusion.^19^ ODI (orientation dispersion index) characterizes the distribution of orientations of neurites within the intra-cellular compartment. It is computed based on a Watson distribution, which describes the dispersion of orientations of cylindrical structures (neurites). It ranges from 0 to 1, where 0 represents highly aligned, organized structures and 1 indicates a highly dispersed orientation of neurites.^20^
